# *k*-Same-Net: *k*-Anonymity with Generative Deep Neural Networks for Face Deidentification †

**DOI:** 10.3390/e20010060

**Published:** 2018-01-13

**Authors:** Blaž Meden, Žiga Emeršič, Vitomir Štruc, Peter Peer

**Affiliations:** 1 Faculty of Computer and Information Science, University of Ljubljana, Večna pot 113, SI-1000 Ljubljana, Slovenia; 2 Faculty of Electrical Engineering, University of Ljubljana, Tržaška cesta 25, SI-1000 Ljubljana, Slovenia

**Keywords:** face deidentification, generative neural networks, *k*-Same algorithm

## Abstract

Image and video data are today being shared between government entities and other relevant stakeholders on a regular basis and require careful handling of the personal information contained therein. A popular approach to ensure privacy protection in such data is the use of deidentification techniques, which aim at concealing the identity of individuals in the imagery while still preserving certain aspects of the data after deidentification. In this work, we propose a novel approach towards face deidentification, called *k*-Same-Net, which combines recent Generative Neural Networks (GNNs) with the well-known *k*-Anonymitymechanism and provides formal guarantees regarding privacy protection on a closed set of identities. Our GNN is able to generate synthetic surrogate face images for deidentification by seamlessly combining features of identities used to train the GNN model. Furthermore, it allows us to control the image-generation process with a small set of appearance-related parameters that can be used to alter specific aspects (e.g., facial expressions, age, gender) of the synthesized surrogate images. We demonstrate the feasibility of *k*-Same-Net in comprehensive experiments on the XM2VTS and CK+ datasets. We evaluate the efficacy of the proposed approach through reidentification experiments with recent recognition models and compare our results with competing deidentification techniques from the literature. We also present facial expression recognition experiments to demonstrate the utility-preservation capabilities of *k*-Same-Net. Our experimental results suggest that *k*-Same-Net is a viable option for facial deidentification that exhibits several desirable characteristics when compared to existing solutions in this area.

## 1. Introduction

In recent years, an ever-increasing amount of image and video data is being recorded, stored or processed worldwide. A key factor that contributes towards this development is the wide-spread availability of personal gadgets, such as mobile phones or tablets, as well as other imaging devices (such as surveillance systems, security cameras and web-cams), which make capturing images and video footage an easy task. While these trends have contributed towards making peoples lives easier in many aspects, care needs to be taken that the captured data are not misused and the privacy of people visible in the imagery is adequately protected.

Privacy protection is especially important, when image and video data are recorded and shared between government entities and other relevant stakeholder, which may be inclined to exploit the data for purposes different from those for which they were recorded. Driver’s licenses, for example, which are used in certain countries as a means of identification, may be exploited for screening purposes against existing watch lists by security agencies; traffic cameras may be used for surveillance purposes; and so on. This kind of function creep can be seen everywhere today and has major repercussions on our privacy and daily lives. While it is necessary to have the technology in place to ensure appropriate levels of security for people, measures also need to be taken to ensure a sufficient level of privacy protection. The outlined issues typically boil down to a single problem: how to perform secure data sharing, while at the same time preventing possible cases of misuse.

**Figure 1:**
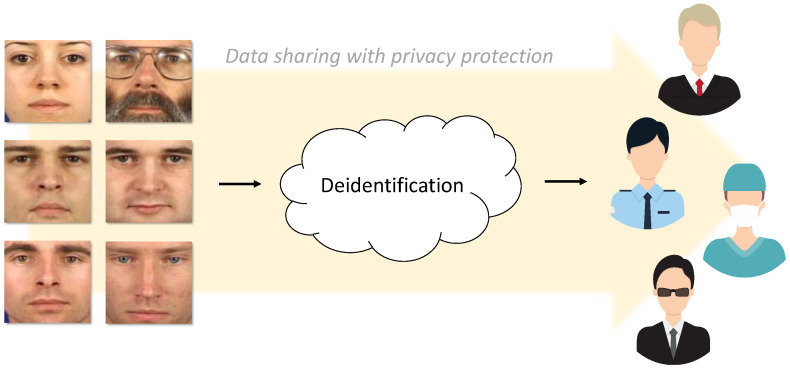
The motivation behind deidentification: to prevent misuse of personal information and ensure privacy protection when data are shared between government entities or other relevant stakeholders, the data need to be appropriately deidentified before being shared.

As illustrated in Figure 1, a common solution to address this problem is deidentification, a process that conceals personal identifiers present in image and video data and thus prevents the recovery and misuse of identity information (e.g., prevents face recognition). Deidentification commonly strives to hide personal information both from human inspection, as well as from automatic recognition techniques and, when applied to image and video data, is typically focused on face deidentification, as the appearance of the human face is by far the most prevalent personal identifier in this type of data.

According to [1,2], early face deidentification techniques mostly centered around naive approaches, such as blacking-out, pixelation or blurring, which are generally considered not to be very effective or suitable for this task. Blacking-out, for example, puts a black patch over the original face image to conceal identity. While this does guarantee anonymity, it also removes all of the non-identity-related information, including characteristics that could be useful for further analysis, but do not rely on identity information. Pixelation and blurring are also considered unsuitable for deidentification, as they are prone to imitation attacks (i.e., parrot attack [1]), where a probe image is simply subjected to the same degradation process as the deidentified images and can then be again recognized reliably (see [3] for empirical examples).

More recent techniques from the literature try to overcome the limitations outlined above and provide formal guarantees regarding the anonymity of the deidentified data, e.g., [4,5,6]. These techniques try to reduce the amount of information that is removed from the imagery, while ensuring upper bounds on the so-called reidentification risk: the risk of reidentifying an image after deidentification. We build on these techniques and present in this paper a novel deidentification approach called *k*-Same-Net. The proposed approach exploits a recent class of generative models, i.e., Generative Neural Networks (GNNs), and combines them with a formal anonymity scheme, i.e., *k*-Anonymity [4]. The generative model is capable of synthesizing natural, realistic-looking surrogate faces for deidentification and parameterizes some of the visual characteristics of the synthesized data. This property makes it possible to retain certain aspects of the original data (i.e., enabling data utility of describable non-identity-related attributes, such as facial expressions, age or gender), while replacing sensitive personal traits with synthetic content. We demonstrate the feasibility of the proposed approach on a closed set of facial images and show comparative results for competing techniques from the literature. We observe that *k*-Same-Net generates convincing surrogate faces without artifacts and is flexible enough to ensure that selected aspects of the data can be retained even after deidentification if needed.

To summarize, the main contributions of this work are:

- We introduce a novel algorithm for face deidentification, called *k*-Same-Net, that is based on a formal privacy protection scheme and relies on GNNs.
- We introduce an additional (proxy) image set that can be used for model training in the deidentification approach, but preserves the formal guaranties that come with *k*-Anonymity-based privacy protection schemes.
- We present an in-depth analysis of the proposed approach centered on reidentification and facial expression recognition experiments.

Note that a preliminary version of this work appeared in [7]. Here, we extend the previous paper by presenting a more comprehensive literature review, providing a more in-depth discussion of key concepts and by adding an extensive experimental section that includes several important experiments aimed at demonstrating the merits of the proposed *k*-Same-Net deidentification approach.

The remainder of the paper is structured as follows. In Section 2, we review the related work including existing deidentification approaches and generative neural networks. In Section 3, we introduce our novel deidentification approach, *k*-Same-Net, that combines generative models with a formal anonymity model. In Section 4, we present qualitative, as well as quantitative experiments exploring different aspects of the proposed deidentification technique and also provide visual deidentification results to highlight the merits of our approach. We conclude the paper with some suggestions for future research in Section 5.

## 2. Background and Related Work

In this section, we describe relevant work from the literature. We discuss deidentification and existing privacy protection schemes, elaborate on existing face deidentification approaches (ad hoc and formal) and present a short overview of generative deep models.

**Deidentification and privacy protection schemes:** A plethora of privacy protection schemes emerged from the need to protect sensitive data mostly in relational databases, where the data reside in tables of columns (known as attributes or quasi-identifiers) and rows (also known as records). Private data holdings, such as hospital data, insurance records or bank-accounting data, are some well-known examples of such data corpora [4].

While large amounts of data are often desired, especially for analytical and data mining purposes, the main problem here is that the processed data may also contain sensitive information, which represents a threat to privacy. Because of that, numerous techniques for privacy-preserving data mining were presented in the literature, which proposed effective mining approaches without compromising privacy. Most of these techniques use some kind of data transformations that result in a trade-off between information loss and privacy. One early example of such techniques is the randomization method, which adds noise to the database entries with the goal of masking the attribute values [8]. A comprehensive review of existing privacy protection schemes can be found in the excellent survey by Aggrawal and Yu [9]. In the remainder of this section, we only focus on formal privacy protection schemes, which are also closely related to the topic of this paper.

Before going further, we note that formal privacy protection schemes, which are at the focus of this work, are currently limited to deidentification of the facial area only. If other biometric traits are present in the data (which is especially the case in video surveillance scenarios), then these schemes are less suitable, since re-identification can be conducted by analyzing the original biometric information beyond the facial area (e.g., gait, posture, clothes, etc.) using either hand-crafted feature extraction methods [10,11,12] or deep learning [13].

In [4], Sweeney introduced the *k*-Anonymity model for deidentifying entries in a relational database. According to her definition, a table adheres to *k*-Anonymity (after deidentification) if and only if each sequence of values for each attribute within that table appears with at least *k* occurrences. Because there are *k* copies of the same sequence in the dataset, the probability of linking an individual sequence to the original is bounded by 1/k. A constrained version of *k*-Anonymity was later proposed by Miller et al. [14] and refined by Campan et al. [15] with the *p*-Sensitive*k*-Anonymity model, which limits the amount of allowed generalization when masking microdata. Recently, new extensions focusing on flexible *k*-Anonymity were proposed in [16,17] to enable the deployment of *k*-Anonymity to other application scenarios by defining a semantic ontology to generate suitable *k*-blocks of data.

Other privacy protection schemes (not based on *k*-Anonymity) have also been proposed in the literature with two of the better known being *L*-Diversity [5] and *t*-Closeness [6]. Machanavajjhala et al. [5] introduced three versions of *L*-Diversity: Distinct *L*-Diversity (which ensures at least *L* distinct values for the sensitive attribute in each equivalence class), Entropy *L*-Diversity (which incorporates entropy and ensures that the entropy of each equivalence class is greater or equal to log(L)) or Recursive (*C*-*L*)-Diversity (which is an alternative definition that ensures meaningful appearances of the most common and the least common attribute values) [5]. *t*-Closeness, proposed by Li et al. [6], on the other hand, is defined as a property that holds for an equivalence class if the distance between the distribution of a sensitive attribute in this class and the distribution of the attribute is not greater than a threshold *t* [6].

**Face deidentification:** Several techniques have been presented in the literature that focus on face deidentification. While some of these build on the formal privacy protection schemes covered in the previous section, many are ad hoc and do not come with anonymity guarantees. We defer the discussion on the first group of techniques to the next section and focus here on the second group of ad hoc methods.

Examples of ad hoc methods include video scrambling [18], image puzzling [19], filtering [20], image foveation [21] or encryption. Face transfer, on the other hand, deals with a similar problem in the field of computer graphics, but is usually not considered in the area of face deidentification, although it could be applicable to this area as well [22,23].

Deidentification is not limited solely to static images, but is applicable to video data as well. Especially with the rise in Unmanned Aerial Vehicles (UAVs), this topic is receiving increased attention in the literature. Works that utilize deidentification on video data exploit JPEG encoding [24] or other compression approaches, e.g., based on the Discrete-Cosine-Transform (DCT) perceptual model [25]. Transform domain scrambling methods were also proposed [18], as well as warping [26], chaos-cryptography [27], blurring [28], pixelation and masking [29].

An example of using foveation in the DCT domain for the purpose of face deidentification was recently proposed by Alonso-Perez et al. [21]. Here, the authors report that DCT-foveation-based deidentification protects the individual’s identity, while preserving gender and expression. The level of privacy-protection ensured by the approach was compared to other naive ad hoc methods (pixelation, blurring, randomization using Gaussian noise and eigen-features) by performing recognition experiments using the (2D)${}^{2}$ PCA-based algorithm at different deidentification strength levels. To evaluate awareness and data utility, the authors used an SVM classifier to classify gender and facial expressions.

A *q*-far deidentification technique is proposed by Samarzija et al. in [30]. The techniques uses Active Appearance Models (AAMs) for deidentification and swaps faces by mapping the texture triangles from the surrogate faces to the target faces. The authors argue that a many-to-many type of deidentification scheme is better than the standard many-to-one scheme (such as *k*-Anonymity), because it does not require knowledge about other identities and the facial replacement looks more natural [30].

Mosaddegh et al. [31] propose a part-based deidentification scheme by aggregating different donors’ face components. The proposed approach uses a bank of face components and Poisson image blending, which mitigates artifacts. Experiments show descriptor-based recognition tests and a qualitative evaluation.

Farrugia et al. [32] presents a reversible deidentification approach for losslessly compressed images, which is compatible with formal privacy protection schemes. Reversible deidentification in this case allows for the recovery of the original content containing private information. Deidentification is achieved using a two-level reversible watermarking scheme. The proposed method is compared with deidentification based on scrambling of DCT coefficients and encryption of pixel values.

Letournel et al. [20] presents a face deidentification method with expression preservation capabilities on the basis of variational adaptive filtering, where the filtering preserves the most important facial regions (i.e., the eyes, the gaze, the lips and their corners). The authors compare the proposed technique with the *k*-Same-Pixel and *k*-Same-M methods. Their evaluation includes software-based recognition tests, as well as the analysis of human recognition performance.

Brkic et al. [33] recently demonstrated face, hair and color deidentification on video data from the ChokePoint dataset. A follow-up work from the same authors using Generative Adversarial Networks (GANs) [34] demonstrated body and face deidentification of people in videos. The authors use human-figure segmentation and replace the segmented contents with synthetic human images with alternative appearance. Although the proposed approach relies on GANs, it is not formulated around any of the formal privacy schemes. Their generative network uses segmentation and replaces the original content with the generated content from the learned data distribution.

***k*-Anonymity and face deidentification:** Formal face deidentification schemes are typically based on the *k*-Anonymity model [4], though many are compatible with related privacy protection schemes, such as *L*-Diversity [5] or *t*-Closeness [6] as well. The *k*-Anonymity model inspired the family of so-called *k*-Same deidentification algorithms, e.g., [1,2,35], which are among the most well-known techniques in this area.

**Figure 2:**
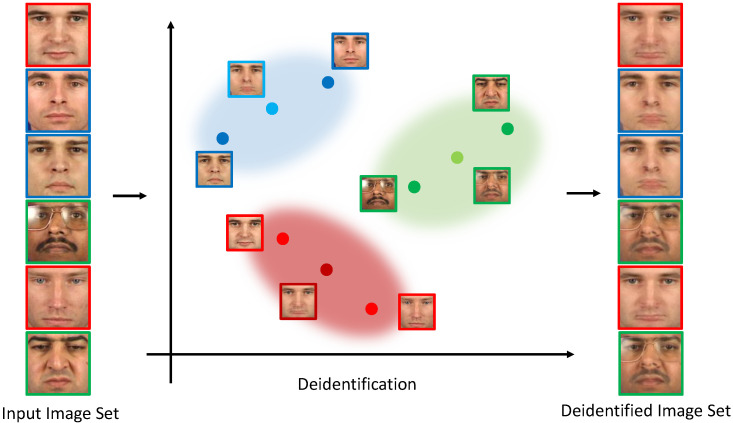
Illustration of the idea behind the *k*-Anonymity mechanisms. The input image set I on the left is mapped to the deidentified image set D on the right. Anonymity is ensured by replacing *k* images from I with the same surrogate image. To preserve some of the information content of the original images, the surrogate images are computed as cluster centroids of the original images in I. The figure shows an example for k=2.

The main idea of the *k*-Same family of algorithms is illustrated in Figure 2. The algorithms take a closed set of *N* images as input $I=\{I_{1},I_{2},\ldots ,I_{N}\}$ and produce a set of *N* deidentified images $D=\{D_{1},D_{2},\ldots ,D_{N}\}$ that cannot be linked to the inputs in an unambiguous manner. Here, anonymity is ensured by identifying clusters of *k* images in the input-image set I and replacing all *k* images of each cluster with the corresponding cluster centroid. As a result, the deidentified image set D contains *k* copies of each computed centroid, and consequently, each image (or centroid) in D bears similarities to all *k* images in the corresponding cluster. The outlined procedure makes it impossible to link any individual from D to the individuals from I with a probability higher than 1/k and provides formal guarantees with respect to the anonymity of the deidentified data. Note that these guarantees apply only if the images in I belong to exactly *N* identities, and hence, each subject in I is represented with a single image only.

The original *k*-Same algorithm, proposed by Newton et al. in [1], operates directly in the pixel space and, therefore, preserves visual characteristics of all *k* images of each cluster in the cluster centroids. The motivation for the algorithm comes from the fact that: (i) replacement of all images in the *k*-sized clusters of I with the same surrogate images ensures anonymity; and (ii) selecting the cluster centroids (of similar faces) as the surrogates minimizes information loss during deidentification. These properties are illustrated in Figure 2, where sample deidentification results for the original *k*-Same approach are presented. As can be seen, the deidentified images still preserve some of the visual information contained in the original images, but also exhibit ghosting effects that appear as a consequence of poor alignment of the images in I.

To address these limitations, an extension of the *k*-Same algorithm was presented by Gross et al. in [36]. The algorithm, named *k*-Same-Model or *k*-Same-M, extends the idea of *k*-Anonymity to Active Appearance Models (AAMs) and applies the deidentification procedure in the AAM parameter space. Because AAMs ensure better alignment between images, the surrogate faces feature almost no ghosting effects and appear more realistic. Nevertheless, some potentially useful information (pertaining, for example, to facial expressions) may still get lost during the deidentification process due to the averaging step.

Many extensions of the above approach have been proposed in the literature focusing mainly on improving the naturalness of the deidentified faces and preservation of as much of the non-identity-related information in the original images as possible. These include the *k*-Same-Eigen [1], *k*-Same-Select [2], *k*-Same-furthest-FET (FET stands for Facial Expression Transfer) [37], *k*-Diff-furthest (“Diff” in method name denotes that this method generates different deidentified face for each of the *k* original faces in a cluster) [38], *k*-SameClass-Eigen [39] and GARP-Face (“GARP” stands for Gender, Age, Race attribute Preservation) [40] algorithms, to name a few. A high-level overview of these algorithms is given in Table 1. For more information on face deidentification, the reader is referred to [41], where a recent survey on this topic is presented.

**Generative deep neural networks:** GNNs represent recent generative models capable of synthesizing artificial, but natural looking images of any object and are, therefore, also highly suitable for the task of deidentification.

Goodfellow et al. [42], for example, proposed the so-called Generative Adversarial Networks (GANs), which combine two competing deep architectures: a generative model that synthesizes artificial images and a second discriminator network that tries to classify the synthesized image as either real or artificially generated. The main idea here is to train the discriminator network as a standard classifier to distinguish between two image sources (real or artificial) and the generative network as a generative model capable of fooling the discriminator network. Back-propagation is used with both the discriminator and the generator network to find how the generator’s parameters should be changed in order to make the generated samples slightly more challenging for the discriminator. Once the training is completed, the generator network outputs images that are indistinguishable from real images for the discriminator and also look visually convincing for human observers.

Mirza and Osindero [43] added conditioning to the original GAN architecture (and called it cGAN), since in an unconditioned generative model, there is no control over the generated data. If the model is conditioned on additional information (such as labels, some part of data or even different modalities), it is possible to direct the generation process. The added information can be fed into both the discriminator and generator as an additional input layer during training. Sricharan et al. [44] recently presented a Semi-Supervised version of cGAN (SS-GAN). Their approach extends the discriminator to a pair of separated discriminators. One of the discriminators is unsupervised and distinguishes only between real and fake images, while the other is trained in a supervised manner (this means it distinguishes between real and fake pairs of images and relies on both the labeled and unlabeled data).

Radford et al. presented a DC-GAN architecture [45], which can be trained in an unsupervised manner. The network takes 100 random numbers (latent variables) drawn from a uniform distribution as input and outputs an image (in this case of size 64×64×3 pixels). As the input variables are changed incrementally, the generated images are too, which shows that the model has learned features to describe how the world looks, rather than just memorizing examples. The experiments demonstrate real-life examples of generated bedroom images and also show promising vector arithmetic operations for visual concepts.

Arjovsky et al. [46] presented improved GANs called Wasserstein Generative Adversarial Networks (WGAN). This work improves the training of the discriminator in such a way that it achieves optimality during training by using a more meaningful loss metric (an approximation of the earth mover distance; also called the Wasserstein-1 metric) before each generator update. The new loss deals with the problem of vanishing gradients and converges to a linear function that gives remarkably clean gradients everywhere [46]. This process is beneficial for the generator due to the higher quality of the provided gradients during training. As reported in this work, none of the experiments showed a collapse of the model for the proposed WGAN algorithm, which shows improved training stability in comparison with the current DC-GAN architecture [45] that the authors used as a baseline.

Nowozin et al. [47] improves on the DC-GAN idea from [45] and shows that *f*-divergences can be used for training GANs. The authors derive GAN training objectives and demonstrate examples with Kullback–Leibler and Pearson divergences. They also simplify the saddle-point optimization procedure from [42] and suggest activation functions for the final layer for the proposed *f*-divergence functions. The use of this upgrade is reported to be limited, since after training, these models cannot be conditioned on the observed data.

Tran et al. [48] presented DR-GAN, which can produce a unified identity representation from one or multiple in-the-wild face images supplied as an input along with information about pose (in the form of pose code). The produced result is generated by virtually rotating the face to an arbitrary pose. This means that the generative part of the network is able to synthesize frontalization of the input face. The discriminative part can be, on the other hand, used as a predictor to infer the identity and pose of a face image.

Karras et al. [49] proposed a novel training methodology based on progressive training and growing of the GAN architecture. This means that both the generator and discriminator start from a low resolution, and during training, new layers are added to increase the image resolution and improve image details. As reported, this process increases the training speed and stabilizes the training process as well. Results show high resolution (1024×1024) generated facial images, and the authors conclude that there is still room for improvement in the micro-structure of the generated images and a long way to true photorealism.

Dosovitskiy et al. [50] introduce a GNN, capable of drawing 2D images of 3D objects given the object style, viewpoint and color. The network architecture used in this work is identical to the standard Convolutional Neural Network (CNN) architectures commonly used for classification, but is turned “upside-down”, which makes it possible to generate synthetic images given high-level information. Thus, instead of learning a classification problem, the authors demonstrate how to generate images from their high-level descriptions. The input parameters consist of three vectors: one-hot encoding of the model identity (which defines the style), azimuth and elevation of the camera position and the parameters of additional artificial transformations applied to the images. The higher network layers first build a shared, high dimensional hidden object representation from the input parameters, whereas the latter layers generate an image and object segmentation mask from the shared hidden representation.

While other generative models have been introduced recently in the literature, e.g., [45,51,52], we build on the work of Dosovitskiy et al. [50] in this paper and devise our *k*-Same-Net algorithm around this class of GNNs. Note, however, that the same idea could be extended to other model architectures as well.

## 3. Neural-Network-Based Deidentification

In this section, we introduce the proposed *k*-Same-Net algorithm. We first present a short overview of the approach, then discuss how deidentification and information preservation is achieved and finally describe the generative part of *k*-Same-Net including its architecture and training.

### 3.1. k-Same-Net Overview

**Figure 3:**
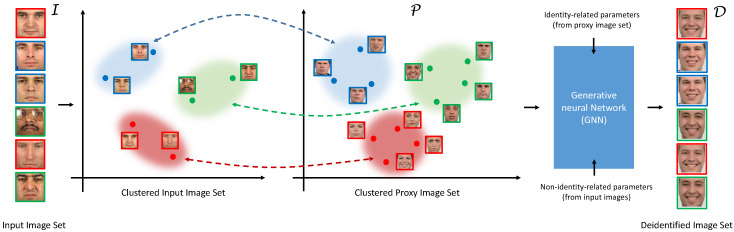
Overview of the *k*-Same-Net deidentification approach. Similar to other *k*-Same algorithms, each image in the input set I on the left is mapped to an image in the deidentified image set D on the right with *k* images from I mapping to the same image in D. The surrogate faces in D are generated by a Generative Neural Network (GNN) that is trained to produce identities from a proxy image set P. Other visual characteristics of the generated surrogate faces (pertaining, for example, to facial expressions, age, gender, etc.) are defined by a set of non-identity-related parameters of the GNN and depending on the application can be easily modified during the deidentification with *k*-Same-Net.

A high-level overview of the k-Same-Net approach is presented in Figure 3. Similar to other algorithms from the *k*-Same family, our approach operates on a closed set of *N* input images I corresponding to *N* distinct identities. The algorithm maps the input set I to a target set of deidentified images D, but unlike existing techniques relies on an additional proxy set of images P to implement the mapping. With our approach, formal anonymity guarantees are again ensured by replacing clusters of *k*-images from I with the same surrogate faces. However, different from competing techniques from the literature (such as [1] or [36]), these surrogate faces are not generated through image or model-parameter averaging, but are synthesized with a GNN and, therefore, bear no visual similarities to the original images from I. More importantly, potentially useful information of the original images is preserved with *k*-Same-Net by exposing a set of appearance-related parameters at the input side of the GNN that affect the visual characteristics of the synthesized images (e.g., facial expression, age, gender, etc.), but not the identity. This approach differs significantly from competing solutions in this field, as useful information is “added back” to the deidentified images (as needed), instead of preserving parts of the appearance of the original images explicitly.

### 3.2. Deidentification with k-Same-Net

Consider a set of *N* input images $I=\{I_{1},\ldots ,I_{N}\}$ belonging to *N* identities and a second (proxy) set of *M* images $P=\{P_{1},\ldots ,P_{M}\}$ corresponding to *Q* identities, where M,Q≥N. Furthermore, assume that our goal is to map the images in I to a target set of deidentified images $D=\{D_{1},\ldots ,D_{N}\}$ in such a way that no relation between the subjects in I and the images in D can be established without ambiguity.

With the original *k*-Same algorithm, clusters of *k* images are generated from I based on image similarities and used to define surrogate faces $D_{i}$ (for i=1,…,N) for deidentification (see Section 2 for details). It is straightforward to extend this approach to proxy clusters defined over P as long as the number of generated clusters for the image sets I and P is the same and a one-to-one correspondence between the clusters is established. Replacing all *k* images of each cluster of I with the same surrogate images achieves so-called *k*-Anonymity, where linking a deidentified image to one of the identities in I is limited to a guess with a success probability of 1/k, regardless of how the surrogate faces are defined (refer to [1] for a formal proof). It is, therefore, possible to compute surrogate faces for deidentification from a proxy image set P that can in general contain an arbitrary number identities, *Q*, as long as the same number of clusters can be computed as for the image set I, i.e., Q≥N. Under these conditions, the proxy set P can be used as the training set for the GNN, and synthetic face images produced by the GNN can serve as surrogate faces for deidentification.

**Figure 4:**
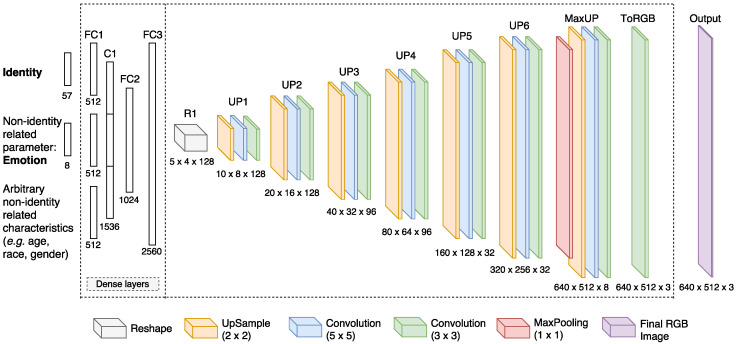
Our GNN architecture is built using fully connected (dense) layers (denoted as FC1, FC2, FC3) and concatenation layer (denoted as C1), followed by six deconvolutional layers (performing upsampling and convolution, from layer UP1 to UP6 as denoted in the figure). Final layers in the network include combination of max pooling and upsampling (denoted as MaxUP) layers and the three-channel convolution (denoted as ToRGB) layer, respectively. Inputs to the GNN are one-hot encoded, which means that many input combinations are possible (e.g., multiple identities). On the output, GNN renders a colored face image through the layer ToRGB as illustrated in the figure.

It needs to be noted that surrogate faces could also be generated based on a single identity from the proxy set P for each cluster of I. However, for practical reasons, we generate the synthetic images of the *k*-Same-Net approach based on multiple (i.e., *k*) identities and, therefore, deal only with artificial identities and not synthetic images of real people present in our training data. We are able to generate images with multiple identities due to the fact that the input parameter, which denotes identity, is one-hot encoded. This means that we enable *k* identities on the Identityinput, and the GNN incorporates these identities into the resulting output image (see Figure 4).

### 3.3. k-Same-Net and Data Utility

While the main goal of deidentification is facilitating data anonymity, the current trend in this area is to also ensure suitable levels of data utility after deidentification. With *k*-Same-Net, we preserve (or better said retain) some of the information content in the original images by probing the input images for certain characteristics and then feed the results of this probing procedure to the GNN to generate images in accordance with the identified (or desired) characteristics. For example, if our goal is to preserve facial expressions after deidentification, we first recognize the facial expressions in the input data and then generate the surrogate faces in accordance with the identified expressions. This procedure is performed for each image separately, so after, deidentification images from the same cluster of I are deidentified with a surrogate face of the same target identity, but may differ in terms of facial expressions. Such an approach allows us to devise selective deidentification schemes tailored towards specific target applications, where only certain visual attributes of the input images are preserved, while others are removed completely.

### 3.4. GNN Architecture and Algorithm Summary

The main component of our *k*-Same-Net approach is the GNN recently proposed in [50] and later extended for face synthesis by M.D. Flynn (https://zo7.github.io/blog/2016/09/25/generating-faces.html). The network consists of a hierarchy of fully-connected and deconvolutional layers and once trained is able to generate synthetic surrogate faces *D* given: (i) information about the *k* identities in the relevant proxy cluster of P encoded in the vector x; and (ii) information about the non-identity-related appearance characteristics of *D* encoded in the appearance-parameter vector y:

(1) D=GNN(x,y).

We present the employed generative architecture in Figure 4, where our network with six deconvolution layers (denoted as UP1 to UP6) is illustrated. It is worth noting that the number of deconvolution layers (consisting of upsampling and convolution operations) is arbitrary and can be increased if larger images need to be generated. On the other hand, the number of layers can also be decreased if a more lightweight model is needed. As it is shown for up-sampling layers, the number of filters decreases deeper into the network while the spatial dimensions are doubled with each deconvolution layer. The max pooling layer (denoted as MaxUP) uses a kernel size of 1×1 for dimensionality reduction (essentially reducing the number of channels) just before the last up-sampling is performed. This is followed by two convolutions, first one uses kernel size 5×5, and second uses kernel size 3×3. The last convolutional layer (denoted as ToRGB) performs 3×3 sized filtering over three channels and produces the final output image. The inputs of the network consist of an identity-encoding vector and high-level parameters encoding non-identity-related characteristics. Although we focus solely on facial expressions (or emotions) in this work, it is trivial to extend the architecture to other characteristics, as well (this is illustrated with the lowest part of the network input in Figure 4).

Training of the GNN requires an appropriately annotated training set with labels spanning all appearance characteristics that need to be altered during image generation. While there is no strict limit with respect to the number of input parameters and appearance variations our GNN can handle, it is necessary that suitable labels exist for all images present in the training set. Note, however, that if the network model is extended, it must be retrained and adapted for each additional input.

A summary of the complete *k*-Same-Net algorithm is given by Algorithm 1.

**Algorithm 1:**
*k*-Same-Net.
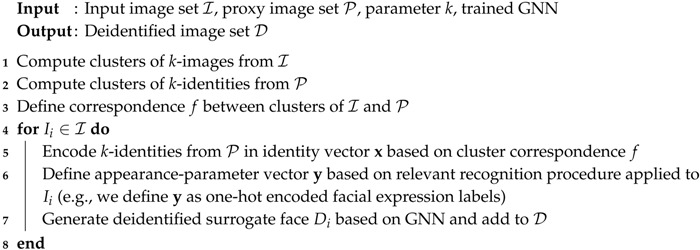


## 4. Experiments and Results

In this section, we present experiments to demonstrate the feasibility of our *k*-Same-Net deidentification approach. We first discuss the datasets used for network training and experimentation and then present comprehensive experiments that illustrate some of the key characteristics of *k*-Same-Net.

### 4.1. Datasets

**Figure 5:**
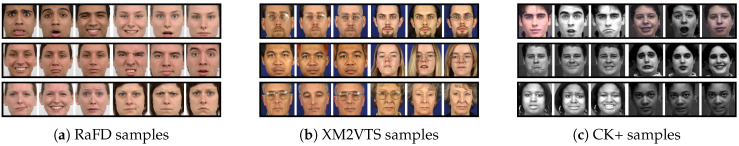
Sample images from the three datasets used for our experiments: (**a**) RaFD [54]; (**b**) XM2VTS [55]; and (**c**) CK+ [56]. We use the RaFD dataset to train our generative network and the XM2VTS and CK+ datasets to highlight the merits of *k*-Same-Net.

For our experiments, we use three publicly available datasets of face images, i.e., Radboud Faces Database (RaFD) [54], The Extended Multi Modal Verification for Teleservices and Security database (XM2VTS) [55] and The Extended Cohn-Kanade Dataset (CK+) [56,57]. We employ the first dataset to train our GNN and the latter two to demonstrate the deidentification and data utility-preservation capabilities of *k*-same-Net. Some example images from the three datasets are shown in Figure 5.

To train the generative network needed for *k*-Same-Net, we use the RaFD dataset [54], which contains high-quality images of 67 subjects with eight different facial expression (i.e., anger, disgust, fear, happiness, sadness, surprise, contempt and neutral) per subject. In RaFD, each subject is captured under three different gaze directions and from five camera angles under all eight facial expressions. From these images, we select the frontal images displaying 57 adult subjects for our training procedure, resulting in a training set size of 456 facial images. The RaFD dataset represents a suitable choice for the training of the generative network of *k*-Same-Net, as it includes aligned high-resolution facial images taken in controlled environments and most importantly because it ships with facial-expression annotations that can be used to demonstrate some of the advantages of the proposed deidentification approach.

We evaluate the deidentification performance of *k*-Same-Net on the XM2VTS dataset [55]. XM2VTS again contains images of good quality taken against a uniform background (see Figure 5b) and features mostly faces with a neutral facial expression. While there is a total of 2360 frontal face images of 295 subjects in the dataset (eight images per subject), we select a subset of the images for our experimentation. In particular, for each experimental run, we experiment with images of 50 subjects to conform with the requirement of *k*-Same-Net that the number of identities in the subject-specific image set I that needs to be deidentified, *N*, is lower than the number of identities, *Q*, in the proxy set P (see Section 3.2 for details). The relatively small number of subjects selected for our experiments makes recognition experiments fairly easy (as shown in the following sections), but at the same time results in a more difficult task for the deidentification procedure, which needs to conceal the identities more effectively to avoid reidentification.

We use the third dataset, CK+ [56,57], to demonstrate the data-utility-preservation capabilities of *k*-Same-Net. Specifically, we show how information on facial expressions can be preserved despite deidentification, thus enabling expression recognition on the deidentified data. The CK+ dataset comprises video sequences of 123 subjects expressing posed and non-posed (or natural) facial expressions/emotions, i.e., anger, disgust, fear, happiness, sadness, surprise and contempt. The sequences start with a neutral expression and proceed to a peak expression (a frame or still image) that is fully coded in accordance with the Facial Action Coding System (FACS) [58]) and associated with an emotion label. We use these peak-expression images for our expression-recognition experiments with the deidentified data. A few examples of these peak images in cropped form are shown in Figure 5c.

### 4.2. Network Training

We train our GNN with selected 456 images (displaying eight emotions from 57 adults) from the RaFD dataset and rely on the implementation of M.D. Flynn (https://github.com/zo7/deconvfaces) for the training. We crop the images to remove some of the background from the images and down-sample them to a manageable size before feeding them to the network for training. We train the network with back-propagation using stochastic gradient descent and the Adam optimizer. We set the batch size to 16, the learning rate to 0.001 and limit the number of epochs to 500 [3]. The training requires approximately 24 h on a desktop PC with 32GB of RAM and a TitanX GPU.

**Figure 6:**
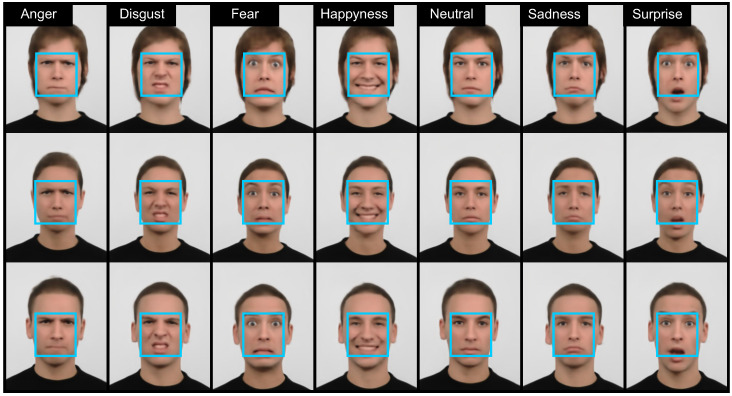
Examples of synthetic images generated by the GNN. The GNN can produce various facial expressions for every identity. Each synthesized face shown is a mixture of *k* identities from the training (or proxy) set with k=2 for the presented examples. Note that all images appear natural and show no visible artifacts (such as ghosting or other non-natural looking distortions).

Once the network is trained, it is able to output realistic, natural-looking facial images of size 640×512 corresponding to the identities from the training data or artificial, non-existing identities and displaying various facial expressions as seen in the examples in Figure 6. The synthesized images shown here are generated by mixing two identities from the training set (or in other words, selecting k=2), so none of the depicted subjects represents a real person. The blue boxes show the image area that represents the surrogate faces, $D_{i}$, needed for deidentification. For the experiments presented in the remainder of the paper, the size and location of the bounding boxes are determined manually in the images, which are simply cropped to include only the facial area.

### 4.3. Qualitative Evaluation

**Figure 7:**
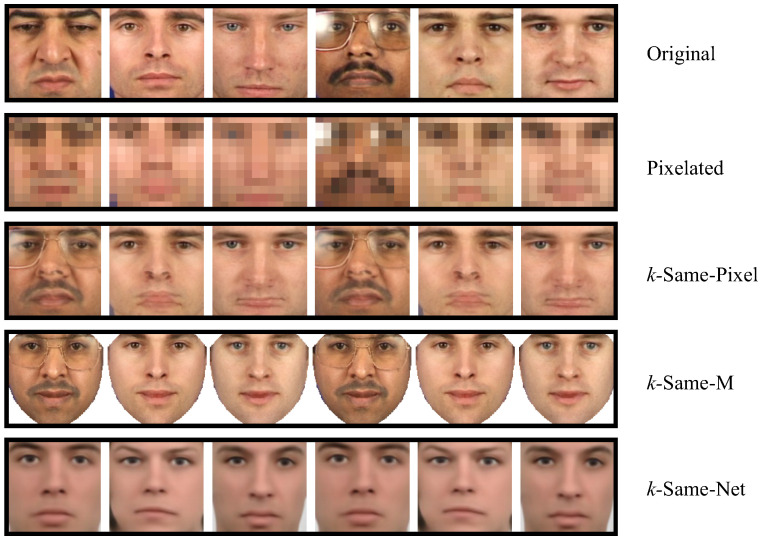
Qualitative deidentification results (from top to bottom): the original images, pixelated images, the *k*-Same-Pixel algorithm (k=2), the *k*-Same-M algorithm (k=2) and the *k*-Same-Net approach (k=2). Note how the deidentification schemes differ in the visual quality of the deidentified images, as well as the amount of preserved information content.

We now present a few illustrative deidentification results based on a selection of images from the XM2VTS dataset. In Figure 7, the top row shows original XM2VTS face images that form our subject-specific input set I. The second row shows the same image set, but deidentified with a naive approach, where the images in I are simply down-sampled (i.e., pixelated) to hide the identities. Note that while some facial features are concealed, a simple imitation attack may suffice to successfully link the naively deidentified images to the originals as shown in [3]. The third row of images shows sample results for the original *k*-Same-Pixel [1] algorithm for k=2. Here, anonymity is guaranteed, and as emphasized in Section 2, it is impossible to link any image from the deidentified image set to the originals with a probability higher than 0.5 (i.e., 1/k=1/2). However, the quality and naturalness of the deidentified images is questionable as artifacts appear due to misalignment of the original images. The fourth row shows images deidentified with the *k*-Same-Model (also referred to as *k*-Same-M) approach using AAMs and k=2. The images here appear more natural than images deidentified through pixelation or *k*-Same-Pixel and exhibit no ghosting effects as the facial texture is mapped to a common shape, but further modifications are needed to ensure that the utility of the data is preserved after deidentification. Our approach, shown in the last row of Figure 7, also comes with anonymity guarantees and again produces natural and realistic deidentification results without any artifacts. These findings are amongst other qualitative pros and cons also summarized in Table 2.

**Figure 8:**
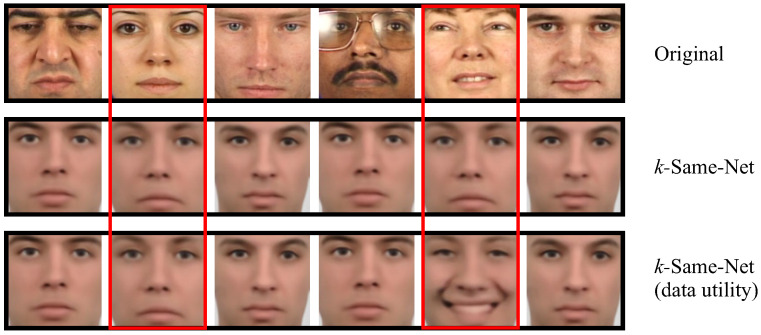
Preserving data utility. The top row shows the original images from the subject-specific input set I; the second row shows images deidentified with *k*-Same-Net without preserving any of the input information; and the last row shows *k*-Same-Net results where the facial expressions of the originals are retained in the deidentified images. Note that the two images that are marked red belong to the same cluster (k=2) and have been deidentified in the last row using the same artificial target identity, but a different facial expression.

A key characteristic of the *k*-Same-Net approach is the possibility of preserving specific non-identity-related information (such as facial expressions) of the original images, while concealing facial identity information with the artificially-generated surrogate faces. Figure 8 illustrates this characteristic on a second subject-specific set of images from the XM2VTS dataset. Here, the top row again shows original images with two new images that were not present in Figure 7 that are highlighted in red. These two images form a cluster and are, therefore, replaced with the same surrogate face images during deidentification, as shown in the second row of Figure 7, where our *k*-Same-Net approach was applied, but the same appearance-parameter vector y (see Equation (1)) was used for all images. The last row shows an oracle-type of experiment under the assumption that the facial expression of the input images is known. The images in this row illustrate how facial expressions can be preserved if needed. The two images from the input set that are marked with red are still deidentified using the same artificial target identity, but now exhibit different facial expressions. Note that under the assumption that the facial expressions cannot be exploited for identity inference, the upper bound on the reidentification performance still equals 1/k in this case.

### 4.4. Recognition and Reidentification Experiments with k-Same-Net

To evaluate the efficacy of deidentification techniques, reidentification experiments are commonly conducted on the deidentified images. The goal of these experiments is to establish the risk of successfully identifying a subject included in the input set I based on the deidentified data in D. The common assumption here is that the identities in I are known, and the task is to link the deidentified images to the subjects in I.

We quantify the reidentification risk through recognition experiments with images from the XM2VTS dataset. We randomly sample 50 identities from the dataset during each experimental run and select one image per subject to include in the subject-specific image set (or probe set) to be deidentified and one image to serve as the gallery. We then perform identification experiments with the constructed probe and gallery sets. We repeat this process five times and report the identification performance in the form of the average rank one recognition rates (Rank-1) and corresponding standard deviations computed over the five experimental repetitions. Most importantly, we conduct the outlined experiments before and after deidentification to assess the level of privacy ensured by *k*-Same-Net.

To cover a broad range of recognition approaches, we select recognition techniques that exploited learned, as well as hand-crafted features for identity inference. Specifically, we include four recent state-of-the-art deep models in the experiments, i.e., AlexNet [59], VGG-Face [60], InceptionV3 [61] and SqueezeNet [62], and two techniques based on dense image descriptors, i.e., LBP (Local Binary Patterns) [63] and POEM (Patterns of Oriented Edge Magnitudes) [64]. We use pretrained publicly available deep models from [65] https://github.com/kgrm/face-recog-eval) and the descriptor-based techniques from the AWE MATLAB toolbox [66] http://awe.fri.uni-lj.si/) for our experiments. With all recognition methods, we simply extract features using the publicly available code and then use the computed feature vectors with the cosine similarity for recognition.

**Figure 9:**
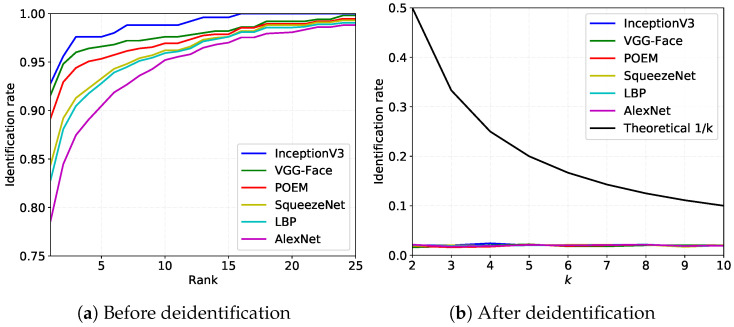
Recognition and reidentification experiments: (**a**) average Cumulative Match Characteristics (CMC) curves of the recognition experiments on the original images of the XM2VTS dataset with five recognition techniques; (**b**) average Rank-1 recognition rates for all tested recognition approaches after deidentification as a function of *k*. The results show that the original identities are correctly determined most of the time on the original images with all techniques considered, while the performance is close to random after *k*-Same-Net deidentification regardless of the value of *k*. The graphs are best viewed in color.

The results of the recognition experiments on the unaltered XM2VTS images are shown in Figure 9a in the form of Cumulative Match Characteristics (CMC) curves and in Table 3 as mean Rank-1 rates with corresponding standard deviations. As can be seen, all reported approaches achieve high recognition rates, which suggests that the original images are relatively easy to identify. Figure 9b shows the recognition performance of the tested recognition approaches after deidentifying the subject-specific image set (the probes) with *k*-Same-Net. Here, the mean Rank-1 recognition rate is plotted against different values of *k*. For reference purposes, the theoretical upper bound on the reidentification risk is also included in the plots. Note that *k*-Same-Net is able to ensure effective deidentification for all values of *k*, as the average recognition performance of all methods is always around 2% regardless of *k*. These results are also supported by the last column of Table 3, where the mean Rank-1 recognition performance and standard deviations are tabulated for *k*-Same-Net deidentification with k=2. All in all, the empirical evaluation suggests that the risk of reidentification for images deidentified with *k*-Same-Net is low, as a random pick from a gallery of 50 subjects is also expected to result in an average rank one recognition rate of 2%. The recognition performance of all tested recognition techniques has, hence, dropped to close to random after *k*-Same-Net deidentification.

### 4.5. Comparison with Competing Deidentification Techniques

We compare our *k*-Same-Net deidentification approach with a number of competing techniques from the literature using the same data and experimental protocol as described in the previous section. We deidentify the subject-specific probe sets using two naive and two formal techniques based on the *k*-Anonymity scheme. From the naive methods we select blurring and pixelation, and from the formal methods we choose: *k*-Same-Pixel [1] (applied in pixel space), *k*-Same-Model [36] (or *k*-Same-M, uses AAMs) for our experiments and compare them with our *k*-Same-Net procedure. We implement the blurring-based deidentification technique by filtering the probe sets with a Gaussian kernel of size 121×121 with σ=15 and the down-sampling-based approach by subsampling the images to 10×10 pixels and upscaling them back to the initial size (320×320 pixels) using nearest neighbor interpolation. To keep the results section uncluttered, we select the two best performing recognition techniques from the previous section for the experiments, i.e., InceptionV3 from the group of methods relying on learned features and the POEM-based approach from the group of methods exploiting hand-crafted features. We again report the average Rank-1 recognition rates on deidentified probe sets for both recognition approaches and all tested deidentification methods.

**Figure 10:**
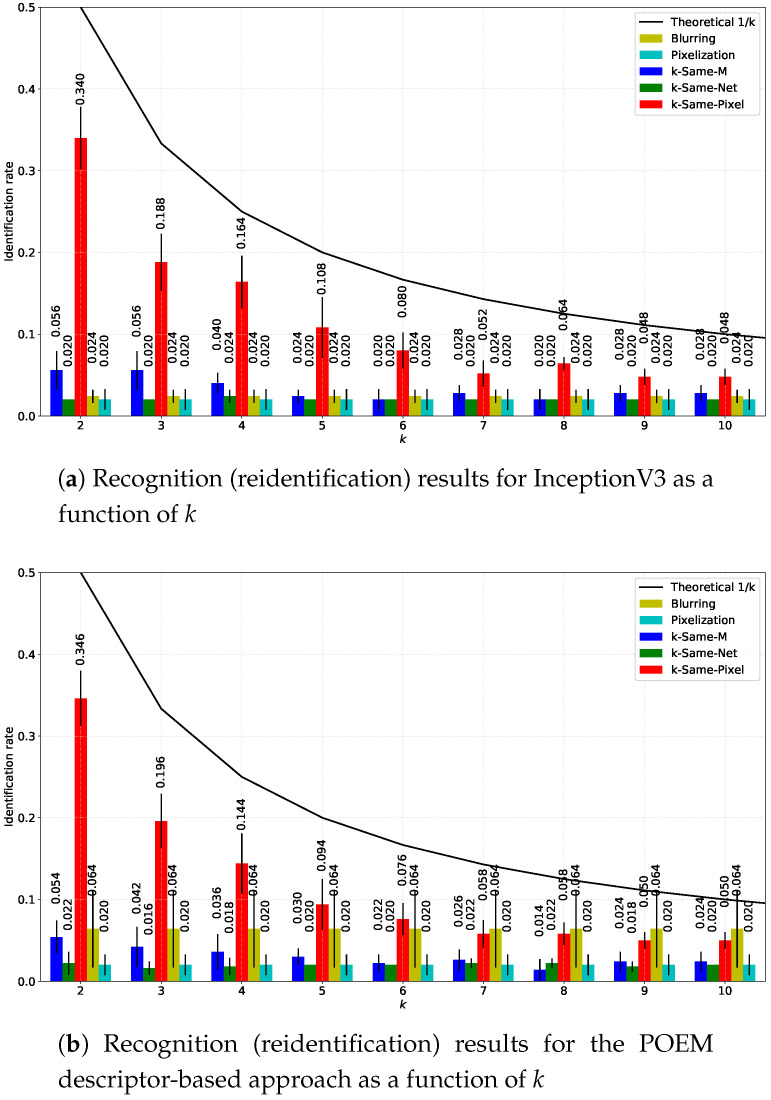
Average Rank-1 recognition (reidentification) rates obtained on the deidentified probe sets over five experimental repetitions with five competing deidentification approaches as a function of *k*: (**a**) results with the InceptionV3 face recognition model; (**b**) results for the POEM-based recognition approach. The results show that *k*-Same-Net is effective and offers high levels of privacy protection compared to competing techniques, while having desirable properties as shown in Section 4.6.

The results for the comparative experiments described above are shown in Figure 10. The performance of the formal methods is presented with respect to the parameter *k*. The upper bound of the reidentification risk (i.e., the guaranteed recognition limit of 1/k) is also displayed to illustrate the theoretical recognition probability of the *k*-Same family of algorithms. In general, all tested deidentification techniques are well below the theoretical upper bound in terms of reidentification performance for both recognition procedures considered. The *k*-Same-Pixel approach ensures the lowest level of privacy protection, but the reidentification performance decreases with higher values of *k*, making the deidentification procedure more effective. The same behavior can be observed for the *k*-Same-M approach, though the reidentification risk is much lower here than with *k*-Same-Pixel. The naive techniques are independent of *k* and offer a good level of privacy protection, albeit at the expense of loosing most of the information content of the images. The *k*-Same-Net approach also ensures a low risk of reidentification for both tested recognition techniques, but the reidentification rate does not seem to be dependent on *k*, as faces from the input set I are replaced with artificially-generated surrogate faces that contain different identities from those being deidentified. As emphasized several times in the paper, the *k*-Same-Net approach still allows preserving certain aspects of the input data after deidentification, which may not necessarily be the case with the competing techniques. We evaluate this characteristic in the next section.

### 4.6. Utility Preservation

We demonstrate the ability of *k*-Same-Net to preserve certain characteristics of the data through facial expression (or emotion) recognition experiments. Our goal with this series of experiments is to demonstrate that with *k*-Same-Net, the deidentified data can still be used for applications where facial-expression information is important, but there is no need to retain identity-related cues. Similar experiments could also be performed for other aspects of the input data by incorporating additional modules into the analytical part of *k*-Same-Net that estimate salient visual characteristics of the input facial images. Due to recent advancements in different areas, such as facial landmarking [67,68,69,70,71,72], facial expression recognition [73,74,75,76,77,78], age estimation [79,80,81,82,83], gender recognition [84,85] and alike [86,87], there are several techniques with open-source implementations available that can be used for this task.

**Figure 11:**
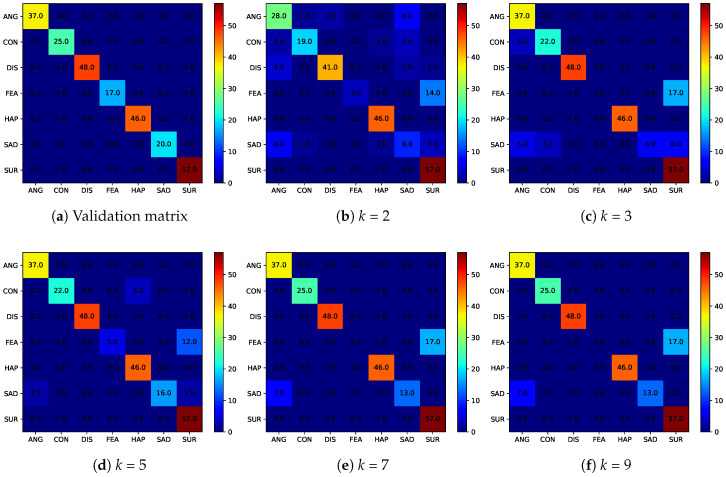
Confusion matrices of the utility-preservation experiments for the DeXpression model: (**a**) the reference matrix generated on the original CK+ images; confusion matrices after deidentification with *k*-Same-Net for: (**b**) k=2; (**c**) k=3; (**d**); k=5; (**e**) k=7; and (**f**) k=9. Note that a high expression recognition performance can still be achieved on the deidentified data.

For the experiments presented in this section, we integrate the DeXpressionmodel [88], a deep convolutional neural network for emotion recognition, into our pipeline and use the expression labels produced by the model to guide the generation process of our GNN. We train the model to recognize the seven established expression classes (i.e., anger, contempt, disgust, fear, happiness, sadness and surprise) on all 327 labeled peak-expression images of the CK+ dataset and the 399 annotated RaFD facial images. To test the performance of the expression recognition model, we again generate five probe sets, each comprised of a single peak-expression image of 50 randomly selected subjects from the CK+ dataset. We apply the trained DeXpression model and compare the predicted expression labels to the annotated ground truth. The results of this experiment are presented in the form of a confusion matrix in Figure 11a. As can be seen, the recognition performance of the trained model is 100%, which is expected, as all images used in this experiment were also included in the training data. The generated confusion matrix serves as the ground truth for demonstrating the utility preservation capabilities of *k*-Same-Net in the following experiments.

Next, we deidentify the five probe sets from CK+ with *k*-Same-Net using different values of *k* and observe how the DeXpression model performs on the deidentified data. Since all of the surrogate face produced by *k*-Same-Net corresponds to artificial identities, none of the subjects produced were included in the training data. From Figure 11b–d, we can see that the expression recognition performance is still very high for all values of *k*. Overall, we achieve an accuracy of 81.2% for k=2, an accuracy of 86.4% for k=3, 92.4% for k=5, 90.4% for k=7 and an accuracy of 90.4% for k=9. It is interesting to observe that the performance peaks at k=5, which also comes with better privacy protection guarantees than the implementations with the lower *k*. This may be explained by the fact that with k=5, the appearance of the generated surrogate faces is already quite similar to an average face. Thus, with limited variations in identity-related appearance, there is more room to better synthesize more explicit facial expressions.

## 5. Conclusions

In this work, we have presented a new approach to face deidentification that combines Generative Neural Networks (GNNs) and a formal privacy protection scheme based on *k*-Anonymity. The approach named *k*-Same-Net utilizes a GNN to generate synthetic face images for deidentification, but enables preservation of selected non-identity-related features (such as facial expressions, gender or age). Our future work will focus on assessing issues related to contextual information, which are known to degrade the effectiveness of deidentification. Since generative models represent the current state-of-the-art in the field of content generation, we expect that more generative deidentification approaches will be seen in the future following a similar methodology.

## Figures and Tables

**Table 1:** Overview of some of the existing formal deidentification techniques based on the *k*-Anonymity scheme (AAM stands for Active Appearance Model, PCA is Principal Component Analysis and FET is abbreviation for Facial Expression Transfer).

Year	Algorithm	Domain	# Assumptions
2005	*k*-Same-Pixel [1]	Pixel	Client specific image set.
2005	*k*-Same-Eigen [1]	PCA space	Client specific image set.
2005	*k*-Same-Select [2]	AAM, PCA space	Need to specify selection criteria
			prior to deidentification.
2008	*k*-Same-Model (or *k*-Same-M for short) [35]	AAM	Model parameters obtained during
	(also known as the (ϵ,k)-map algorithm)		fitting are not unique due to ambiguities.
2013	Driessen–Durmuth’s algorithm [53]	PCA space, Gabor wavelets	Not achieving very strong *k*-Anonymity;
			human recognition is still possible.
2014	*k*-Same-furthest-FET [37]	AAM, PCA space	Neutral emotion not available explicitly;
			FET not satisfying *k*-Anonymity;
			efficacy experimentally proven.
2014	GARP-Face [40]	AAM	Utility-specific AAMs for
	(Gender, Age, Race Preservation)		ethnicity, gender, expression.
2015	*k*-Diff -furthest [38]	AAM	Distinguishable client specific image set.
2017	*k*-SameClass-Eigen [39]	PCA, LDA space	Depends on LDA classifier accuracy;
			may fail on unknown faces.

**Table 2:** Qualitative pros and cons comparison of the evaluated deidentification methods (based on the opinion of the authors).

Type	Method	Pros	Cons
Naive	Pixelization [1]	Easy to implement. Training not required.	No formal privacy guarantees.
attack	Blurring [1]		
Formal	k-Same-Pixel [1]	Easy to implement.	Ghosting effect, visible artifacts.
	k-Same-M [35]	Visually convincing.	Training required.
	k-Same-Net (ours)	Visually convincing. Offering data utilization.	

**Table 3:** Recognition performance over five repetitions of recognition experiments with images form the XM2VTS dataset before and after *k*-Same-Net deidentification. Six different recognition approaches are included in the comparison exploiting learned, as well as hand-crafted features. The results suggest that the *k*-Same-Net deidentification approach ensures a high-level of privacy protection.

Feature Type	Method	Rank-1 (μ±σ)—Before deid.	Rank-1 (μ±σ), k=2—After deid.
Learned	Inception V3 [61]	0.928±0.035	0.020±0.000
	VGG-Face [60]	0.916±0.040	0.016±0.012
	SqueezeNet [62]	0.844±0.055	0.019±0.016
	AlexNet [59]	0.786±0.131	0.021±0.015
Hand-crafted	LBP [63]	0.828±0.094	0.021±0.016
	POEM [64]	0.892±0.055	0.019±0.015
